# Urine exosomes as biomarkers in bladder cancer diagnosis and prognosis: From functional roles to clinical significance

**DOI:** 10.3389/fonc.2022.1019391

**Published:** 2022-09-20

**Authors:** Nicholas Lee, Ashan Canagasingham, Mohit Bajaj, Ramesh Shanmugasundaram, Anthony Hutton, Joseph Bucci, Peter Graham, James Thompson, Jie Ni

**Affiliations:** ^1^ St George and Sutherland Clinical Campuses, School of Clinical Medicine, Faculty of Medicine & Health, UNSW Sydney, Kensington, NSW, Australia; ^2^ Department of Urology, St George Hospital, Kogarah, NSW, Australia; ^3^ Cancer Care Centre, St George Hospital, Kogarah, NSW, Australia

**Keywords:** exosome, bladder cancer, liquid biopsy, biomarker, diagnosis

## Abstract

Bladder cancer is one of the top ten most common cancers and top ten causes of cancer death globally. 5-year survival rates have decreased in Australia from 66% to 55% in the past three decades. The current gold standard for diagnosis is cystoscopy. However, cystoscopies are an invasive and health-resource intensive procedure which has sub-optimal sensitivity for flat lesions such as CIS (carcinoma in situ) and low specificity for differentiating inflammation from cancer - hence requiring biopsies under anesthesia. Frequent and life-long surveillance cystoscopy is required for most patients since there are high rates of progression and local recurrence in high-risk non-muscle invasive cancer (NMIBC) as well as poor outcomes associated with delayed detection of muscle-invasive bladder cancer (MIBC). There is an unmet need for a non-invasive test to provide better discrimination and risk-stratification of bladder cancer which could aid clinicians by improving patient selection for cystoscopy; enhanced risk stratification methods may guide the frequency of surveillance cystoscopies and inform treatment choices. Exosomes, which are nano-sized extracellular vesicles containing genetic material and proteins, have been shown to have functional roles in the development and progression of bladder cancer. Exosomes have also been demonstrated to be a robust source of potential biomarkers for bladder cancer diagnosis and prognosis and may also have roles as therapeutic agents. In this review, we summarize the latest evidence of biological roles of exosomes in bladder cancer and highlight their clinical significance in bladder cancer diagnosis, surveillance and treatment.

## Introduction

Bladder cancer was the tenth most common cancer globally, with an estimated 573,278 new diagnoses and 212,536 deaths, in 2020 (1). Bladder cancer mainly affects the elderly population, and the age-adjusted incidence of bladder cancer is significantly higher in males compared to females (34.2 vs 8.5 per 100,000) (2). Muscle-invasive bladder cancer (MIBC) invades into or past the muscularis propria, whilst cancer confined to the urothelium or lamina propria is classified as non-muscle invasive bladder cancer (NMIBC). NMIBC accounts for 80% of newly diagnosed bladder cancers and is further sub-divided into low-risk (low-grade, non-invasive papillary tumors) and high-risk (high-grade papillary tumors with/without invasion and/or CIS) groups with additional intermediate and very high-risk groups recommended by some researchers (3). NMIBC has 90% 5-year overall survival and > 95% cancer-specific survival rates (4) however the rate of recurrence and progression can be as high as 70% (4, 5) and 75% (4), respectively, in the high-risk group. Life-long surveillance is required for high-risk patients due to the high rates of recurrence which, if not detected early, can progress to MIBC by the time symptoms develop. Delayed detection of recurrence at an advanced stage (T2 or higher) is associated with worse overall survival rates (6) - with 5-year overall survival rates below 50% (7). The need for frequent surveillance cystoscopy (e.g. every 3 months for 2 years then every 6 months for 5 years then annually lifelong in high-grade NMIBC) has made bladder cancer one of the most resource-intensive malignancies to manage (8). Therapeutic options for localized bladder cancer have not significantly progressed for the past two decades, which are limited to transurethral resection of bladder tumor (TURBT) and intravesical therapy for NMIBC (9), and cystectomy/radiotherapy +/- systemic therapy for MIBC (10).

Early detection at a curable stage, accurate risk-stratification, timely treatment and adequate surveillance are key to improving long-term survival for bladder cancer patients. Cystoscopy is the current gold standard for bladder cancer diagnosis but is an invasive procedure which suffers from poor sensitivity (58-68%) for flat lesions such as CIS and non-papillary tumors (11). Urine cytology is non-invasive and has a high specificity (95%) when reported as consistent with high-grade malignancy (12), however it is more often reported as atypical or suspicious which only confers a PPV of 6-39% (13) and 47-63% respectively (14). Cytology also has poor sensitivity (37%), particularly for low-grade tumors (15). Furthermore, bladder cancer is associated with a high tumor mutation burden and multiple studies have identified a wide range of distinct molecular signatures of bladder cancer (16–22). This genetic heterogeneity presents a challenge in the use of genomics for detection and risk-stratification of bladder cancer but, if solved, genomics may improve selection for: diagnostic and surveillance cystoscopy, intra-vesical therapy, early cystectomy in high-risk NMIBC, chemo-/immuno-therapy or novel targeted therapies in MIBC and nodal/distant metastatic disease.

Exosomes are nano-sized extracellular vesicles (EVs), carrying cell-specific cargoes of proteins, lipids and nucleic acids, which are present in almost all body fluids and released by a variety of cell types by exocytosis (23). Recently, exosomes have garnered much research interest as they can transfer cargos to recipient cells - forming complex networks that connect tumor cells with tumour cells, and tumor cells with the tumor microenvironment (24). There has also been research surrounding the use of exosomes in bladder cancer diagnosis and prognosis as they may potentially be a non-invasive, economic, and convenient “liquid biopsy” tool with high sensitivity and specificity (25). Exosomes appear to be abundantly present in urine, in which the lipid bilayer protects genomic and proteomic cytoplasmic contents from degradation by urinary acidity and enzymes (26). Furthermore, since exosomes and their cargoes may provide robust information regarding the molecular landscape of bladder cancer, they may be able to stratify disease which could optimize treatment pathways and improve patient outcomes (27). In this mini-review, we discuss the biogenesis and cargoes of exosomes, summarize the latest evidence of their biological roles and highlight the clinical significance of urine exosomes in bladder cancer diagnosis, surveillance and treatment.

## Exosomes

Exosomes, approximately 30-150 nm in diameter, are small EVs secreted from cells and have a bilipid membrane which protects their cargo from external influence, particularly the hostile acidic environment of urine (28). This property increases the feasibility and practicality of analyzing the cytoplasmic contents of exosome cargoes, as a source of tumor genomic and proteomic information, which may in turn provide a unique advantage over cellular and cell-free genomic tests for diagnostic use. On the interventional front, exosomes may be engineered or “repackaged” and potentially have therapeutic applications such as their use as a vector in gene therapy or a vehicle to deliver chemo-/immuno-therapeutic agents (29).

The biogenesis of exosomes occurs within cells *via* the endocytic pathway - with several key processes: the formation of endocytic vesicles, the generation of multi-vesicular bodies (MVBs), and the release of exosomes (30, 31). The inward budding of endosomal membranes results in the formation of intraluminal vesicles (ILVs) (32). MVBs, which are late endosomes, accumulate ILVs within their endosomal lumen (33). One fate of MVBs is their fusion with the plasma membrane and exocytosis, which releases ILVs, now termed “exosomes”, into the extracellular space (34).

Exosomes contain nucleic acids, proteins, lipids, and metabolites; however, their exact contents vary with the type and physiological state of the parent cells (30). The ESCRT (endosomal sorting complexes required for transport) family of proteins play an important role in the unique enrichment of these exosomal cargoes compared to the parental cells (35). Furthermore, the selective sorting of exosome cargoes has also been found to occur *via* novel ESCRT-independent pathways such as *via* tetraspanin-mediated e.g. (CD9, CD63, CD81) or lipid-raft mediated mechanisms (31). Lipids such as cholesterol, sphingomyelin, and phospholipids are enriched in exosomes and have attracted attention due to novel discoveries regarding exosomal lipid-based biomarkers (36) and their impacts on pharmacokinetics (37). It has been shown that there is also selective sorting of both non-coding RNAs (38), including microRNAs (miRNAs), long-coding RNAs (lncRNAs), and circular RNAs (circRNAs); as well as messenger RNA (mRNA) in exosomes (31). RNA-binding proteins and membrane proteins have been found to regulate the selective sorting mechanisms of miRNAs in exosomes - and it has also been demonstrated that several disease states, such as cancer and heart disease, have associated effects on miRNA expression in exosomes (39, 40).

## Functional roles and clinical significance of exosomes and their cargoes in bladder cancer

### Functional roles in cancer and bladder cancer

**Figure 1:**
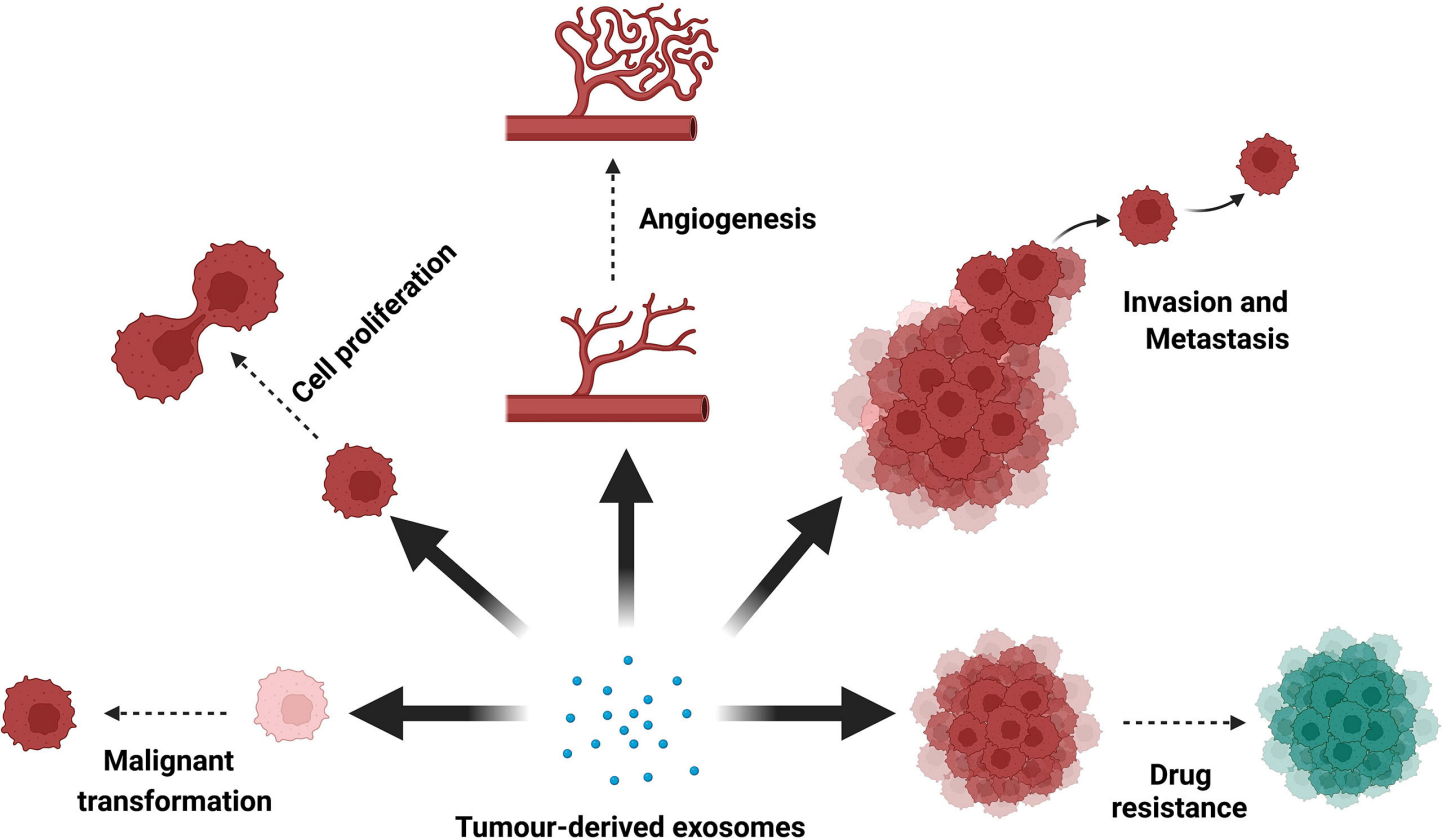
Biological functions of tumor-derived exosomes. Tumor-derived exosomes have important roles in tumorigenesis, proliferation, angiogenesis, invasion, metastasis and drug resistance in almost all cancer types.

Exosomes act as intercellular messengers that ferry active biological molecules accumulated from parent cells to target cells. The transfer of genomic, transcriptomic, proteomic and metabolomic information to target cells may promote changes in metabolism and phenotype (41). In general, tumor cells produce a greater number of exosomes compared to healthy cells (42), making them an ideal candidate for cancer detection. The oncogenic properties of cargoes in tumor-derived exosomes have been shown to aid in tumor development, invasion, metastasis and drug resistance (43) (**Figure 1**).

In bladder cancer, exosomes have been shown to promote cell proliferation, migration, and invasion (44); angiogenesis (45); and malignant behaviors (46). Lin et al. found that exosomal miR-21 promoted bladder cancer progression by polarizing tumor-associated macrophages *via* PI3K/AKT pathway (47). It was also shown that bladder cancer EVs could facilitate the malignant transformation of non-malignant cells by activating endoplasmic reticulum stress-induced unfolded protein response and inflammation *in vitro* (48). In the tumor microenvironment, cancer-associated fibroblasts-derived exosomes could directly transport miR-148b-3p into bladder cancer cells, which was responsible for increased metastatic behaviour and drug (paclitaxel and doxorubicin) resistance *in vitro* and *in vivo* (49).

### Clinical significance of exosomal cargoes as biomarkers for bladder cancer

Exosomes contain a variety of biologically functional molecules that capture a real-time snapshot of the heterogeneity of the entire tumor. In addition, exosomes are stable, abundant and accessible in almost all types of body fluids (42). Exosome biomarkers may improve the current standard of care by identifying patients with aggressive forms of bladder cancer - allowing for optimal management and treatment decisions, reducing the frequency of surveillance, and potentially forming the basis of new treatments. **Table 1** summarizes some key findings in the clinical utility of exosome cargoes in the diagnosis and prognosis of bladder cancer.

**Table 1:** Clinical significance and performance of exosomal cargoes as biomarkers in bladder cancer.

Urine exosome biomarker/s	Biomarker type	Clinical significance	Performance	Reference
miR-21-5p	miRNA	Diagnosis of negative urine cytology bladder cancer.	AUC = 0.900 Sensitivity: 75.0% Specificity: 95.8%	(50)
miR-6124: miR-4511 ratio	miRNA	Discriminate hematuria from bladder cancer.	Sensitivity (in patients with gross hematuria): 94%	(51)
lncRNA UCA1-201	lncRNA	Single biomarker with diagnostic potential.	AUROC: 0.73 (vs normal samples), 0.93 (vs total controls)	(52)
lncRNA UCA1-201, lncRNA UCA1-203, MALAT1, LINC00355	lncRNA	Potential diagnostic panel.	AUC = 0.96 Sensitivity: 92% Specificity: 91.7%	(52)
LASS2 and GALNT1; ARHGEF39 and FOXO3	mRNA	Cancer vs non-cancer differentiation.	LASS2 and GALNT1 in cancer patients; ARHGEF39 and FOXO3 in non-cancer	(53)
lncRNA HYMA1, LINC00477, LOC100506688 and OTX2-AS1	lncRNA	Potential biomarker for high-grade MIBC.	lncRNAs enriched in high-grade MIBC vs control	(54)
lncRNAs (MALAT1, PCAT-1 and SPRY4-IT1)	lncRNA	Improved diagnostic value compared to urine cytology.	AUC: 0.813 Sensitivity: 62.5% Specificity: 85.0%	(55)
PCAT-1 and MALAT1	lncRNA	Association with NMIBC recurrence-free survival.	Correlation between RFS of NMIBC with PCAT-1 and MALAT1	(55)
APOA1, CD5L, FGA, FGB, FGG, HPR, and HP	Protein	Several proteins which could serve as bladder cancer grade discriminators	AUC values ranged from 0.762 to 0.830	(56)
TACSTD2	Protein	Potential role in bladder cancer diagnosis	AUC = 0.735 p = 0.02	(56)

#### Exosomal RNAs as biomarkers in bladder cancer

The recent discovery of nucleic acids in urine exosomes has emerged as promising diagnostic biomarkers for bladder cancer. In a comparative study, Perez et al. evaluated the mRNA expression in five bladder cancer patients and six non-cancer patients and found that LASS2 and GALNT1 were present in cancer patients, while ARHGEF39 and FOXO3 were only present in non-cancer patients (53). Another study (n=60) carried out by Matsuzaki et al. found that urinary exosomal miR-21-5p was able to differentiate urothelial carcinoma patients even with negative cytology (AUC=0.9, sensitivity, 75.0%; specificity, 95.8%) (50). Piao et al. found that the expression ratio of miR-6124 to miR-4511 was significantly higher in the bladder cancer groups than in patients with hematuria or pyuria (sensitivity, 91.5%; specificity, 76.2%) and the sensitivity even increased to 94.0% in patients with gross hematuria (51). These findings are significant as they have identified biomarkers that can distinguish which patients with hematuria should undergo a full work-up, with greater sensitivity and specificity compared to cytology.

As bladder cancer is a heterogeneous disease characterised by a high mutation burden, several studies highlighted the importance of integrated molecular profiles rather than single gene tests which may be abnormal in some tumors but not others. Using RNA sequencing, Huang et al. identified an RNA panel consisting of three mRNAs (KLHDC7B, CASP14, and PRSS1) and two lncRNAs (MIR205HG and GAS5) that is able to distinguish bladder cancer patients from healthy volunteers (AUC=0.924, 95% CI, 0.875–0.974), and the expression levels of these five RNAs were correlated with clinicopathological features (57). Similarly, a study conducted by Yazarlou et al. (n=108), found that the expression levels of an exosome lncRNA panel (UCA1-201, UCA1-203, MALAT1 and LINC00355) had high sensitivity and specificity in differentiating urothelial carcinoma from normal samples (92% sensitivity and 91.7% specificity) (52). However, although biomarker panels have higher diagnostic performance compared to single or dual biomarkers, there are issues in their translation to clinical implementation as the cost, complexity, and convenience of the test are important factors to consider and they require multiple external validations in independent studies across a range of populations.

Exosomal biomarkers have also been researched as prognostic markers for bladder cancer but are largely in infancy. Andreu et al. demonstrated that urinary exosomal miR-375 was a biomarker for high-grade bladder cancer while miR-146a could identify low-grade patients using a microarray platform (58). Other studies found that urinary exosomes from patients with high-grade bladder cancer were enriched in lncRNA HYMA1, LINC00477, LOC100506688, OTX2-AS1 (54) and TERC (59). Recently, Zhan et al. established that upregulation of exosomal lncRNA PCAT-1 and MALAT1 was associated with poor recurrence-free survival (RFS) of NMIBC, with PCAT-1 overexpression being an independent prognostic factor (55). These lines of evidence further support the feasibility and utility of urinary exosome biomarkers for bladder cancer risk stratification, to inform use and intensity of intra-vesical and radical treatment options for high-risk NMIBC, and to guide personalized treatment for MIBC. There is currently a large-scale prospective cohort study (n = 3000), in its recruitment stage, evaluating the clinical performance of a urine exosome-based test in the diagnosis of bladder cancer in hematuria patients, and the identification of recurrent disease in bladder cancer patients (NCT04155359).

#### Exosomal proteins as biomarkers in bladder cancer

There have been several studies that investigated the association between the urinary exosome proteome and bladder cancer. Smalley et al. (60) identified the upregulation of several proteins in urinary exosomes of bladder cancer patients compared to healthy individuals (n=9). Furthermore, five of the nine differentially expressed proteins (NRas, EPS8L1, EPS8L2, Mucin 4, and EH Domain-containing Protein 4) are implicated in the epidermal growth factor receptor (EGFR) pathway that is associated with worse prognosis in bladder cancer (61). Using quantitative proteome profiling, protein makers TPP1, TMPRSS2 and FOLR1 were consistently detected and highly upregulated in urinary exosomes derived from the bladder compared to those derived from the ureter. The study also revealed that a distinct population of exosomes released from the bladder might promote distant recurrence through metabolic rewiring, even after apparent complete downstaging (62). More recently, the same group further confirmed in 10 cT2 bladder cancer patients that despite the absence of detectable tumor, the entire bladder released exosomes that contribute to metastasis, regardless of sampling site, and highlighted the need for early radical cystectomy in cT2 bladder cancer (63).

Chen et al. (56) examined urinary exosome proteins in bladder cancer patients and identified that the concentrations of 24 proteins changed significantly compared to the control group, with AUC values ranging from 0.702 to 0.896. Moreover, they found that concentrations of TACSTD2 in urinary exosomes had 6.5-fold higher expression in bladder cancer patients compared to control patients, which has high potential as a novel biomarker for early diagnosis and prognosis for bladder cancer.

### Exosomes as therapeutic targets

Apart from the applications in diagnosis and prognosis, novel therapeutic approaches involving exosomes may also be a possibility. Methods of altering exosome biogenesis, delivery or cell uptake could be investigated and potentially used to control the detrimental effects of tumor-derived exosomes. There are currently no studies of therapies targeting cancer-derived exosomes in bladder cancer per se, although studies have suggested that EV-targeting antibodies or antagonists showed therapeutic and chemo-/immuno-sensitizing potentials in breast (64), pancreatic (65, 66) and colorectal (67) cancers.

There is also interest in utilizing exosomes as a therapeutic vector. Due to their size, ability to cross biological barriers and autologous nature, exosomes could be packaged with pharmacological drugs or tumor-suppressive RNAs that could alter the phenotype of malignant cells (64). Early-phase clinical trials testing EV-based cancer therapy have been completed (68, 69), confirming their capacity to produce anti-tumor effects in patients, and warrant the feasibility of further large-scale investigations. Phase I clinical trials demonstrated the ability of dendritic cell-derived exosomes to exert natural killer cell effector functions in patients, however, a phase II clinical trial evaluating its effectiveness as maintenance immunotherapy on non-small cell lung cancer did not reach its primary endpoint (69). Several other clinical trials are currently underway, testing exosomes as a delivery vehicle for anti-tumor drugs (NCT01294072) or small interference RNAs (NCT03608631).

## Challenges and future perspectives

Exosomes and their cargoes have generated increasing research interest over the last decade. Investigations have demonstrated promising results regarding their use as biomarkers in the diagnosis and risk-stratification of bladder cancer. The efficient and accurate isolation, quantification and profiling of exosomes are crucial for biomarker discovery. In the current standard workflow, these steps are performed separately. While the techniques are well-established, they are often laborious, costly and time consuming, limiting their application in clinical settings. Recently, several “lab-on-a-chip” fluorescent (70), magneto-electrochemical (71), nanoplasmonic (72) and cationic lipoplex nanoparticle (73) technologies have been developed for detection of proteins and miRNAs in exosomes from biological fluids. However, they either require pre-processed clinical specimens or involve intricate fabrications. Future research is warranted to overcome these challenges and ultimately establish the value of a single integrated platform in a clinically validated study to enable point-of-care diagnostics. It has also been demonstrated that exosomes could be used as therapeutic agents in various types of cancers. Exosomes have unique advantages to current therapeutic agents, such as their high drug release stability, bio-compatibility and penetration of biological barriers; a greater understanding of their biological mechanisms and further clinical studies will aid in the development of exosomes for cancer therapy (74).

In the current clinical landscape for bladder cancer, there are several key areas where exosome-based biomarkers could provide benefit: (i) diagnosis; (ii) frequency of surveillance; (iii) type and intensity of intra-vesical treatment; (iv) selection for radical treatment in high-risk NMIBC; (v) selection for neo-/adjuvant chemotherapy and/or immunotherapy; and (vi) monitoring of disease progression. As stated previously, early diagnosis and personalized surveillance of bladder cancer could improve long-term survival, cost and quality of life outcomes. Additionally, from a clinical and health system perspective, the accurate stratification of lower-risk patients could reduce the need, or frequency, for cystoscopy thus reducing the burden on patients and healthcare systems.

## Author contributions

NL, JT and JN conceived the project. NL, AC, MB, RS and JN prepared the initial draft. NL prepared the figure and table. AH, JB, PG and JT revised the manuscript. All authors have approved the submitted version of the manuscript.

## Funding

This work was supported by Cancer Care Centre Research Trust Fund, St George Hospital.

## Conflict of interest

The authors declare that the research was conducted in the absence of any commercial or financial relationships that could be construed as a potential conflict of interest.

## Publisher’s note

All claims expressed in this article are solely those of the authors and do not necessarily represent those of their affiliated organizations, or those of the publisher, the editors and the reviewers. Any product that may be evaluated in this article, or claim that may be made by its manufacturer, is not guaranteed or endorsed by the publisher.
